# Skin-Related Neglected Tropical Diseases (Skin NTDs)—A New Challenge

**DOI:** 10.3390/tropicalmed4010004

**Published:** 2018-12-25

**Authors:** Roderick J. Hay, Kingsley Asiedu

**Affiliations:** 1 The International Foundation for Dermatology, London W1P 5HQ, UK; 2 Department of Control of Neglected Tropical Diseases, World Health Organization, 1202 Geneva, Switzerland; asieduk@who.int

Medical teaching has emphasised over many years the uniqueness of disease states, valuing the rare skills on which the art of diagnosis is based and the intricacies of individual patient-centred management. Yet with the growing appreciation of the public health dimensions of illnesses and the shrinking of the world due to modern travel, coupled with a massive expansion in the availability of, and access to, data, it has become increasingly important to recognise that good health outcomes are often best achieved by pooling expertise and implementing collective actions. The concept of Neglected Tropical Skin Diseases (Skin NTDs) is an example of this approach. Skin NTDs are diseases that present with lesions on the skin surface which may, in turn, provide not only practical clues to the diagnosis but also a greater understanding of disease through investigation, such as mapping, as well as management by identifying common pathways for therapeutic interventions [1,2]. Adopting strategies based on this idea opens access to a reservoir of skills and knowledge that shows how one disease can contribute to a better understanding of others; the integration and exploitation of areas of commonality are both key to initiatives in public health. As an example, the use of mass drug administration (MDA) has also highlighted what NTDs, and their management pathways, have in common rather than what separates them. The use of ivermectin, for example, in control programmes for lymphatic filariasis and onchocerciasis has produced unintentional, but major impacts on the prevalence of other common diseases from soil helminth infections to scabies [3]. This has provided an incentive to pursue the control of these diseases in other regions, as was seen in the recent clinical trial of ivermectin in Fiji for the control of scabies [4]. The use of azithromycin for both yaws and trachoma is a further example [5].

A patient’s skin is accessible and easy to examine, after basic training, by any health care worker. It is therefore a common starting point for disease recognition. It is equally important to appreciate that skin diseases in general are very common, accounting for between 10 and 30% of all health worker/patient encounters depending on location, climate, genetic predisposition, underlying health and local prevalence of transmissible disorders. Therefore, while the skin may provide an entry point for the recognition of NTDs, it is also the focus of a number of very common conditions. Navigating this milieu involves recognising the clues that may lead a) to the identification of NTDs usually through examination and further simple investigations coupled with b) the provision of simple schemes for the management of the most common skin disorders. The development of a simple framework for accomplishing this strategy is an achievable goal. For instance, the World Health Organization (WHO) has recently published a training guide for the recognition of NTDs and common skin problems [6]. 

This issue takes this approach a step further by exploring the use of other techniques such as distance consultation using Telederm or Whatsapp, and simple training pathways for providing support to field workers as well as the introduction of a downloadable app for the recognition of skin diseases. Problems in diagnosis remain where there is a range of clinical manifestations that may lead to a single diagnosis (e.g., mycetoma), or where the presentation of disease states is camouflaged by inappropriate treatments such as cheap and easily available corticosteroids. The operation of community-based schemes for diagnosis and management is explored further as is the potential for new diagnostic interventions. The use of these interventions, adapted however to local conditions, is critical, an issue which is also explored in this series. In addition, the skin is highly visible to the patient or family member, and any disease that affects it is both noticeable and will have an impact on personal and social wellbeing. Changes to the skin often re-enforce feelings of social isolation and stigma experienced by patients with NTDs, and addressing these diseases must form a central plank of any control strategy.

Identifying a common ground is critical for the successful control and management of a number of neglected diseases, particularly for reasons of practicability in implementing operations in the field. Grouping some of these together as skin NTDs will advance this cause. This strategy also produces different impacts on different diseases in different ways. For example, in the case of leprosy, despite the introduction of post-exposure prophylaxis, the identification of cases through recognition of the signs on the skin remains at the heart of effective control. The same is true of other neglected diseases from mycetoma to Buruli ulcer. Ensuring that patients can be identified is also critical for those diseases that are amenable to mass drug administration, because the detection of the remaining cases will be a key element of the task of completing elimination. There will be areas where MDA cover has been incomplete or where finding “missed” cases forms a key to preventing resurgences in the future or recognizing the emergence of drug resistance. Seizing a common ground in the management of disease disability is also crucial as, for instance, the care, rehabilitation and protection of peripheral limbs is a key strategy for leprosy, podoconiosis, mycetoma and lymphatic filariasis as it is in diabetic foot [7,8,9]. These arguments for an integrated approach extend even further through interdependence and the promotion of community education, the relief of stigma, disease mapping, as well as research and training. 
